# Factors influencing the uptake of evidence in child health policy-making: results of a survey among 23 European countries

**DOI:** 10.1186/s12961-021-00786-y

**Published:** 2021-11-07

**Authors:** Kinga Zdunek, Denise Alexander, Peter Schröder-Bäck, Michael Rigby, Mitch Blair

**Affiliations:** 1 Department of Health Education, Faculty of Health Sciences, Medical University of Lublin, 1 Chodźki Street (Collegium Universum), 20-093 Lublin, Poland; 2 Section of Paediatrics, Imperial College London, London, UK; 3 School of Nursing and Midwifery, Trinity College Dublin, The University of Dublin, Dublin, Ireland; 4 Department of International Health, Faculty of Health, Medicine and Life Sciences, Care and Public Health Research Institute (CAPHRI), Maastricht University, Postbus 616, 6200 MD Maastricht, The Netherlands; 5 School of Social, Political and Global Studies and School of Primary, Community and Social Care, Keele University, Keele, UK; 6 Department of Primary Care and Public Health, Imperial College London, London, UK; 7 Lavender Hill, 6 Carrighill Lower, Calverstown, Co., Kildare, R56 DT91 Ireland; 8 Department of Paediatrics, St Mary’s Medical School Building, Imperial College London, London, W2 1PG UK

**Keywords:** Evidence-informed policy, Barriers and facilitators to using health research, Primary child healthcare

## Abstract

**Background:**

The ability to successfully transfer knowledge across international boundaries to improve health across the European Region is dependent on an in-depth understanding of the many factors involved in policy creation. Across countries we can observe various approaches to evidence usage in the policy-making process. This study, which was a part of the Models of Child Health Appraised (MOCHA) project assessing patterns of children’s primary care in Europe, focused on how and what kind of evidence is used in child health policy-making processes in European countries and how it is applied to inform policy and practice.

**Method:**

In this study, a qualitative approach was used. The data were analysed in accordance with the thematic analysis protocol. The MOCHA project methodology relies on experienced country agents (CA) recruited for the project and paid to deliver child health data in each of 30 European countries. CAs are national experts in the child health field who defined the country-specific structured information and data. A questionnaire designed as a semi-structured survey instrument asked CAs to indicate the sources of evidence used in the policy-making process and what needed to be in place to support evidence uptake in policy and practice.

**Results:**

In our data we observed two approaches to evidence usage in child health policy formulation. The scientific approach in our understanding refers to the so-called bottom-up initiatives of academia which identify and respond to the population’s needs. Institutional approaches can be informed by scientific resources as well; however, the driving forces here are governmental institutions, whose decisions and choices are based not only on the population needs but also on political, economic and organizational factors. The evidence used in Europe can also be of an external or internal nature. Various factors can affect the use of evidence in child health policy-making. Facilitators are correlated with strong scientific culture development, whereas barriers are defined by a poor tradition of implementing changes based on reliable evidence.

**Conclusions:**

Focusing on the facilitators and actively working to reduce the barriers can perceivably lead to faster and more robust policy-making, including the development of a culture of scientific grounding in policy creation.

**Supplementary Information:**

The online version contains supplementary material available at 10.1186/s12961-021-00786-y.

## Background

There are many different patterns of children’s primary care in Europe, which leads to many different policies and different health outcomes of children throughout the European Union (EU) and European Economic Area (EEA) countries [1]. One aim in improving children’s public health is to understand why some countries have successful policies in children’s health and good patterns of child health through effective services, and others struggle to achieve the same results. The successful transfer of knowledge across international boundaries to improve health across the European Region requires an in-depth understanding of the many aspects involved in policy creation and identifying relevant transferability criteria [2]. An important factor in achieving this is understanding national influences on policy-making.

The use of evidence in the process of policy formulation is one of the prerequisites for successful policy-making. Evidence, understood as “findings from research and other knowledge that may serve as a useful basis for decision-making in public health and health care” [3, 4], helps in implementing solutions which are responsive to the population needs and are acceptable to the population. However, we should remember that “Evidence alone does not make decisions” [5]. Firstly, it informs expert opinions, but it is not in itself expert opinion. Secondly, to be convincing, it must be based on research that uses standardized and systematic methodologies. Finally, it has to be of high quality, and therefore trustworthy [5].

It is important, therefore, to investigate the impact of context in the process of policy-making and also in the use of evidence to support the policy. Bowen and Zwi highlight an important aspect of the context within which evidence is used: “A key challenge to public health is to better contextualize evidence for more effective policy-making and practice” [6].

Across countries, there are various approaches to evidence usage in the policy-making process. For example, in Australia [7], a “strong institutional foundation for nurturing evidence-based policy (EBP) capacities” underpins policy development [8]. The United States has a long tradition of evidence-based policy-making as it “has been the major global location for policy analysis and evaluation professionals, both within government and in other policy-relevant sectors” [8]. In the United Kingdom attempts to “develop a coherent approach to policy development, championing EBP as a major aspect of the increased policy capability and the fresh thinking required by a reformist government” were undertaken in the early 2000s [8]. Hasanpoor et al. stressed that evidence-based management “has been slowly adopted by healthcare managers in the USA, the United Kingdom and Canada” but a “remarkable gap exists between this ideal scenario and the status quo” [9].

Even though awareness of evidence-based solutions is increasing, there is still a significant need for the development of standardized tools and identification of appropriate measures. Key global and European institutions are undertaking actions to support evidence-based policy-making. Developed by the European Commission, the “Knowledge for policy” portal provides support “to make informed policy decisions regarding the design of research and innovation policies or strategies” [10], with the goal of “[bridging] the science–policy gap by bringing together evidence for policy from scientists across Europe, to policymakers across Europe” [11]. WHO has published a manual with guidance which helps to understand what evidence-based policy is and provides technical support in the process of preparing, developing and conducting it [12].

In this study, we focused on the kind of evidence that is used in decision-making processes and how it is implemented to inform policy and practice in child health policy-making in Europe [13]. This research is part of the Models of Child Health Appraised (MOCHA) project which aimed to assess the varied patterns of children’s primary care in Europe [14, 15]. “A key objective for MOCHA was firstly to describe the primary care systems in detail and their components and to appraise them from a number of different viewpoints, professional, public (including parents, children and wider community), political and economic lenses” [16]. One of the goals of the project was to identify optimal patient-centred and prevention-oriented primary child healthcare models. The conditions for implementation of the alternative models, transferability and preferences of the public were tested at the macro, meso and micro levels.

This transferability analysis was supported by an assessment of the culture of evidence-based policy-making. Our aim was to establish the extent of use of evidence, and the types of evidence that influence and underpin policy-making for children’s health and healthcare. We explored how policy decisions are made in the different countries, and the most common patterns of evidence used at the national health policy level in each MOCHA project country. To this end, we asked two research questions of national correspondents of countries within the EU and EEA Europe:

- How is evidence used in child health policy formulation?
- What are the facilitators of and barriers to evidence usage in child health policy formulation?

## Methods

### Ethics

The MOCHA project was funded as an integrated research programme by the European Commission's Directorate-General (DG) for Research and Innovation. The research protocol, as enshrined in Grant Agreement 634201, recognized the interlinking work between partners to holistically address the core research topic, and was explicit in the use of country agents (CA) as field-level contributors, drawing on local sources but not involving personal data. The nature of a dynamic integrated study is such that each interdependent component, one of which is reported in this paper, cannot be individually reviewed in an informed way at the institutional level. The Ethical Committee of the European Commission's DG for Research and Innovation Horizon 2020 programme was seen as the supranational ethical review committee, assessing the project in whole and as the sum of the parts. The strand of work reported here was explicitly included on page 10 of Grant Agreement 634201, and the CA methodology on pages 128–129 of that document, which was scrutinized and approved by the Ethical Committee of the European Commission's Research and Innovation Directorate as a prior requisite to the project being funded.

### Study design

We used a qualitative approach in this study. The data were analysed in accordance with the thematic analysis protocol proposed by the experienced qualitative researchers Braun and Clarke [17]. We conducted our research in a constructionist manner, which means that we sought to “theorise the socio-cultural contexts, and structural conditions” [17] of the evidence-based usage in child health policy-making in European countries.

### Questions and questionnaire

The MOCHA project methodology relies on the experience of CAs in each of 30 EU and EEA countries. CAs were national experts in the child health field who defined country-specific structured information and data. The CAs were recruited, based on their knowledge and professional experience, by the project coordinators (project principal investigator, project deputy principal investigator, research coordinator) and were paid to deliver child health data for each of the 30 European countries studied. Their contributions ensured that the findings of this paper were based on detailed and local indigenous knowledge. In line with the overall methodology developed by the MOCHA project, CAs were encouraged and trusted to gain a range of opinions within the country prior to distilling it into the formal answer to the question. The answers from CAs were not personal opinions, and regular meetings with CAs confirmed that they were totally opposed to expressing personal opinions and always identified local robust sources according to each topic before providing the final answers to MOCHA researchers.

To analyse evidence-based approaches to child health policy in Europe, a questionnaire designed as a semi-structured survey instrument was created (Additional file 1). The questions were formulated by topic lead researchers (the authors), approved by the project coordination team of scientists, and validated by the project’s external advisory board (EAB), which ensured scientific and professional validity. The EAB comprised members nominated by European medical, paediatric and policy bodies, WHO Regional Office for Europe, United Nations Children's Fund (UNICEF) Innocenti research centre, and civil society groups, and is published elsewhere [18].

The questionnaire was sent by the MOCHA research coordinator via email to each CA. CAs had 1 month to provide the answers. The electronic responses were gathered by the MOCHA research coordinator and sent to the researchers responsible for the analysis.

Specifically, CAs were asked to indicate the sources of evidence used in the policy-making process and what needed to be in place to support evidence uptake in policy and practice by identifying up to three new child-oriented policies developed within the past 3 years (either currently in operation or soon to be implemented) that affect child primary healthcare service provision. Examples of such policies which were put forward included vaccination policy, services for children with special needs, approaches to dealing with abused children, mental health promotion in primary care and changes to out-of-hours service availability. Other types of policy might concern general issues involving the provision of and access to care, human resources, or the organization of the system of financing. For each of their three selected examples, CAs were asked to (a) identify the policy, (b) characterize evidence used in formulating the policy and (c) point out what needed to be in place to support evidence uptake in policy and practice in their countries (Additional file 1). The questions were asked in an open format.

We were aware of the consolidated criteria for reporting qualitative research (COREQ) approach [19], but it could not be applied directly to our study because the researchers did not engage directly in the field, but rather through the CAs, in order to obtain topical material in each national language. However, our methods map to all the key COREQ principles which are appropriate to this multinational study.

### Survey

The data collection was carried out between July 2017 and January 2018. The 30 CAs received the questionnaire by email from the research coordinator. CA representatives of 23 countries—all of the EU member states at that time except Belgium, France, Germany, Latvia, Luxembourg, the Netherlands and Sweden, plus the EEA countries Iceland and Norway—responded to the questionnaire. Lack of responses to the questionnaire by seven CAs was the result of work overload at that time, and overriding personal issues.

In total, 34 policy topics were identified which were related to child primary healthcare in European countries and described in terms of evidence used.

### Data analysis

Braun and Clarke [17] define thematic analysis as a tool which enables the researcher to identify, analyse and report patterns (themes) within data. They offer a six-phase guide, which we followed:

1. Familiarizing with the data in order to mark some ideas for coding. At that stage, we pre-reviewed the data and incorporated it into the computer software for qualitative analysis (NVivo 11).
2. Generating initial codes by creating a list of ideas about what is in the data that results in the creation of meaningful groups of data. We identified our data as latent (interpretative). For coding the data we used computer software. This helped us to tag and name selections of text within each data item. The data were coded phrase by phrase.
3. Identification of themes which, as recommended by Braun and Clarke [17], concentrate on “sorting the different codes into potential themes, and collating all the relevant coded data extracts within the identified themes” [17]. In our research we identified the themes in an inductive, bottom-up way, which enabled our approach to avoid being driven by the pre-existing coding frame.
4. Reviewing and refining themes, which led us to create a thematic map.
5. Defining and naming themes.
6. Producing the report in the form of the presented paper.

### Data availability

As previously explained in other works [20] under Horizon 2020 funding rules, the European Commission requires all data to be accessible to the greatest degree possible, while also recognizing the potential conflict between openness and confidentiality. To that the MOCHA team have added the importance of comprehension, as source data cannot be interpreted without a clear understanding of the method of acquisition. The MOCHA data contain no patient information, but may contain other personal or institutional data such as source of a commentary. The MOCHA project has therefore resolved that source data will be curated on the MOCHA website, and will be accessible via the principal or other partners through a curator function, so that data relevant to any enquiry can be supplied, and redaction effected, but also contextualization given. The curator in this context is an informed data manager who will discuss the objective and meaning of the question so as to supply the required data in a contextually informed manner; the role is analogous to a research librarian facilitating bibliographic searches.

## Results

The results presented below are based on the CAs’ responses obtained. From the characteristics of the evidence usage in child health policy formulation, we identified several structural approaches.

### Types of evidence used in child health policy formulation

**Fig. 1:**
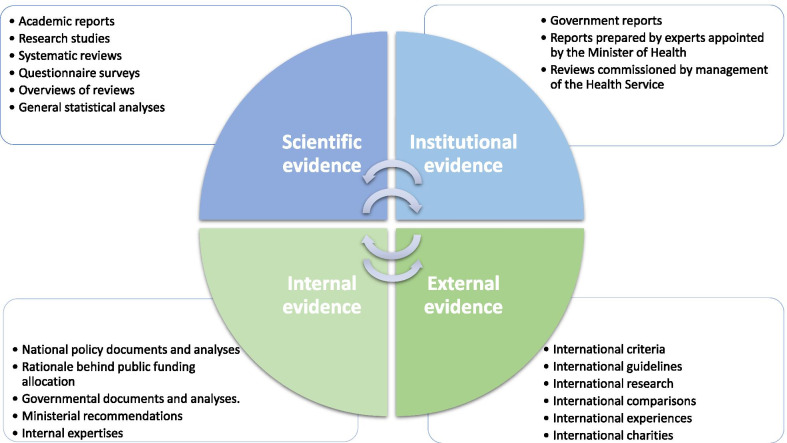
Evidence used in child health policy-making process

In our data we observed two approaches to evidence usage in child health policy formulation (Fig. 1). On the one hand, the responses of CAs show that the evidence used was based on experts’ reports/research studies and developed by professionals, and on the other hand, the baseline for formulating new policy was other policy documents or reports produced by administrative units. The scientific approach in our understanding refers to so-called bottom-up initiatives of academia which identify and respond to the needs of the population. Institutional approaches can be informed by scientific resources as well; however, the driving forces here are governmental institutions, whose decisions and choices are based not only on the population needs but also on political, economic and organizational factors.

Additionally, we observed that the data used in the child health policy formulation can be of an external or internal nature. External evidence refers to international resources and exchange of good practices between European countries, and internal evidence indicates domestic data produced nationally.

To understand our data in wider context we refer to the final MOCHA report where we explain how to bring MOCHA lessons to provided services [15, 21].

### Scientifically produced evidence

Scientific data is a vital tool in the process of child health policy-making, and often it is the baseline for the development of national and international recommendations and guidelines. Examples emerged from Austria, Greece and Spain, with subjects covering child health policy and primary healthcare reform.

The Greek reform of primary healthcare and implementation of urgent amendments under the mandate of the Ministry of Health and other provisions used a report by a working committee of academic professors and medical doctors as a stimulus. The document “Basic Principles, Positions and Proposals for the Development of Primary Health Care in Greece”, was created in 2015 in order to “identify problems of the primary health care system in Greece and provide suggestions on how to rectify them based on evidence” (Greek CA).

Several CAs reported the use of research in the process of developing child health policy in their countries. For example, in Austria, when the new law on health reform implementation was enacted, the strong voice of paediatricians was noticeable in the discussion on the new law, as they criticized the inadequate consideration of primary healthcare for children (Austrian CA). Systematic reviews, questionnaire surveys and overviews of reviews were carried out when the new mother and child health passport (MKP) was developed (Austrian CA).

The development of the “National Strategic Plan for Childhood and Adolescence 2013–2016 (II PENIA)” in Spain, a “strategic plan that seeks out the ‘need to formulate a comprehensive strategy for children based on the principles and provisions of the Commission on the Rights of the Child’ is part of a global and international strategy for children and adolescents” (Spanish CA). The main principles of II PENIA are the best interest, non-discrimination, development of child potential and the right to quality education and participation (Spanish CA). To gain a better understanding of the situation of children, general statistical analyses were conducted to “continue the development of statistical compilations or publications about the ‘situation of children in Spain’ or ‘Children in figures‘ on a regular basis in order to develop series” (Spanish CA) and to “offer new statistical information on issues that affect children between 0 and 18 years, disaggregated by sex, age, disability status and habitat” (Spanish CA). Additionally, the Spanish CA reported that a statistical bulletin of measures for child protection and a statistical bulletin of measures imposed on young offenders were in use.

### Institutionally produced evidence

CAs reported several key sources of evidence from governmental institutions and organizations. Examples from Denmark, Poland and Ireland show that the institutional initiatives to create evidence- and experience-based policies can be relevant here.

In Denmark, the amendment of the law on the use of coercion in psychiatry was the result of a political field of interest and discussion among the political spokespersons. The Danish CA stressed that “the government's psychiatric committee completed a report that highlighted some points of impacts and recommendations in the field of psychiatry, and the possible need for changes in the Danish Mental Health Act” (Danish CA). The amendment “was based on a report, prepared by the government's psychiatric committee. It was, therefore, more experience-based rather than evidence-based. There was little focus on whether there was good evidence for the amendment of the law” (Danish CA).

In Poland, a new plan of coordinated care within primary care was preceded by several studies. In recent years, four main analyses have been undertaken in the field of primary healthcare in Poland, summarized in a report prepared by a team of experts appointed by the Minister of Health to develop strategies for systemic solutions in the area of primary care. The reports were titled “Analysis of Primary Health Care in Poland and proposal of systemic changes” 2016; the National Health Fund research report, “Primary Health Care—the potential and its usage” 2016; the audit report of the Supreme Audit Office, “Functioning of Primary and Outpatient Specialist Care Financed from public funds” 2014; and Ernst & Young Research Report “Optimization of the Polish System of Financing Primary Health Care” 2012 (Polish CA).

In Ireland, debate emerged on Medical Card eligibility as part of the National Medical Card Unit Strategic Plan 2016–2018. The Irish CA explained that “a Medical Card allows people to access Family Doctor or GP [general practitioner] services, community health services, dental services, prescription medicine costs, hospital care and a range of other benefits free of charge. The provision of a Medical Card is based on a means test. However, if a person’s income exceeds the threshold, but their individual circumstances place them at risk of ‘undue financial hardship', including, for example, a child with a chronic illness, they may be granted a Discretionary Medical Card which provides the services as outlined above” (Irish CA). The evidence used in the national public debate comprised the findings of two reviews which were commissioned by the Health Service Executive to inform the development of the policy (Irish CA).

### External evidence

Austria, Croatia, Denmark, Poland and the United Kingdom looked beyond their own boundaries to gain ideas on screening schedules, amblyopia, healthy living, oral health, anti-bullying environment and primary care coordination. External evidence seemed to be called upon most when countries were already aware they needed new ideas.

The policy described by the Austrian CA had the goal of “evaluation, qualitative advancement, attractiveness and increased use as an instrument for the early screening and promotion of children”. To reach these objectives, the Ministry of Health launched an interdisciplinary process aimed at the contemporary development of the MKP, which has operated since 1974 and is regularly amended. The planned implementation of the new mother–child screening, scheduled for 2018, was a further development of the MKP. It developed the assessment of each item of the new screening concept based on international criteria and guidelines (WHO, German guidelines, etc.) (Austrian CA). Additionally, systematic reviews, budget impact analyses, questionnaire surveys, overviews of reviews and guidelines were carried out. The issues were also discussed during conferences such as the European Forum for Evidence-based Health Promotion and Prevention in June 2017. In the second case described by the Austrian CA, international guidelines were used when the health reform legislation (Gesundheitsreformumsetzungsgesetz 2017 - GRUG 2017) establishing primary healthcare centres was prepared. “Research regarding the evidence of PHC [primary healthcare] as well as international comparisons of different models of PHC were the basis for the recommendations for the Austrian model” (Austrian CA).

The Croatian CA identified three areas of child health. Two of these, the national children’s Amblyopia plan and the national “Healthy Living” programme were directly linked with child health. The national strategic plan to improve oral health, despite not being exclusively focused on children’s oral health, devoted most of the document and almost all of the activities to a focus on children. In achieving this, an important role was played not only by professional pressure but also by international comparisons.

Experiences from other countries were used in Denmark in implementing the “Act on the Educational Environment for Students”. This law was an anti-bullying policy that “gives children the right to complain to state authority, if the school/municipality does not take adequate action to stop bullying” (Danish CA). The evidence on how bullying affects children's health in the short and long term was used in formulating these recommendations. In particular, the experiences from Norway and Sweden were taken into account, as both of those countries have for many years had similar complaint boards against bullying.

In Poland, the implementation of a model of coordinated care in primary healthcare was discussed. In accordance with the newly proposed model of coordinated care, all care provided to a child should be performed by a team of health professionals. Such team should consist of a physician, nurse, and midwife and in the future a health educator and dietician as well. In the process of preparing the reform, its authors referred to scientific studies and international experiences such as a report prepared by a team called to develop strategies for systemic solutions in the area of primary care, appointed by the Minister of Health—“analysis of Primary Health Care in Poland and proposal of systemic changes” 2016.

The need for a policy on female genital mutilation (FGM) was raised in the United Kingdom. The United Kingdom CA stressed that the grassroots movement that raised the profile of FGM comprised London-based community groups such as Forward. A Member of Parliament (MP), Ann Clwyd (MP for Cynon Valley in Wales), took up the cause and lobbied parliament to make FGM illegal in the United Kingdom, as well as to develop a policy to aid healthcare professionals. She added that “evidence collected by Forward and other community groups who lobbied for FGM to be made illegal in the United Kingdom was used”. Data from international charities were also used in campaigning by Ann Clwyd (United Kingdom CA).

### Internal evidence

Other policies drew more upon national material. This seemed to apply primarily to situations when a current policy was being reviewed for effectiveness, as opposed to looking for new ideas, and occurred in the Czech Republic, Finland, Norway and Romania concerning patient rights for children, coercion in psychiatry, child and family service reform, free medical service eligibility, early intervention in families, legal framework for vaccination and sustaining healthy eating. These examples have a common theme of seeking to fine-tune and improve established policies; external evidence might be used as a secondary benchmark, as in one example.

In Norway, when the policy “Patients’ rights for children”, aimed at empowering children and youngsters, was introduced, “the white paper documented cases reminding of root cause analysis as in patient safety” (Norwegian CA). A unified political approach to safeguard children was presented in the major newspapers and television.

When a Finnish project of the Ministry of Social Affairs and Health and the Ministry of Education and Culture—called "The programme to address reform in child and family services”—for the years 2016–2018 was created, an analysis of both the shortcomings and the strengths of the current situation was conducted. “Shortcomings in the management and integration of the services and in encountering clients are well documented. Services in many health and social care settings are scattered to many sectors and are mainly organized based on administrative branches. At the same time, there are numerous examples of successful developments of new service structures and services. The programme plan combines these results” (Finnish CA).

Denmark’s policy on early intervention for vulnerable families (Tidlig indsats for sårbare familier) is a political agreement. It was funded by a public funding pool, the Satspuljemidler, which specializes in supporting social, health and work-related dimensions, particularly the most vulnerable in society. “Prior to the implementation of the policy, a mapping was carried out to determine who the vulnerable and disadvantaged children and families are, what initiatives the municipalities have for the families, and the detection and categorization methods used in the different municipalities” (Danish CA).

In Romania, the decline in vaccine coverage and an increase in measles outbreaks, as well as a lack of some of the vaccines in the national vaccination scheme, prompted the Ministry of Health to establish a legal framework for vaccination and the purchase of vaccines. The evidence used in the process of policy development was based on government documents and analyses. “Evidence for drafting the new Law on Immunization was presented in the Exposure of Motives published on the 10.04.2017 by the Ministry of Health” (Romanian CA). This report also relies on “examples from 11 of the 28 EU countries, where vaccination is compulsory and children cannot be admitted in public education institutions without certificates of immunization” (Romanian CA).

In the Czech Republic a campaign called “Prepare your snack yourself” was initiated. It aimed to change the eating habits of Czech school children and involved children in preparing their own nutritionally balanced snacks. This was a plan to combat the childhood obesity trend. An association called “Healthy eating in schools” composed of parents, students, counsellors, nutritional therapists and other professionals on healthy lifestyle wanted to involve more than 150 schools in this initiative. This led to the formulation of the “Snack Decree” and methodical recommendations of the Ministry of Education, Youth and Sports and the Ministry of Health, and nutrition recommendations from the Czech Ministry of Health for evaluation of food provided by school canteens/cafeterias/restaurants. The evidence used in the preparation of the documents was based on the opinions of nutrition health experts of the Ministry of Health and National Institute of Public Health and in consultation with children's nutritionists from the Czech Medical Association (Czech CA).

### Evidence uptake in policy and practice: facilitators and barriers

CAs were asked to describe the elements which facilitate and impede the approach to evidence-based practice in their country. This led to the identification of facilitators and barriers in the evidence uptake in child health policy and practice in European countries. Several patterns were observed.

**Fig. 2:**
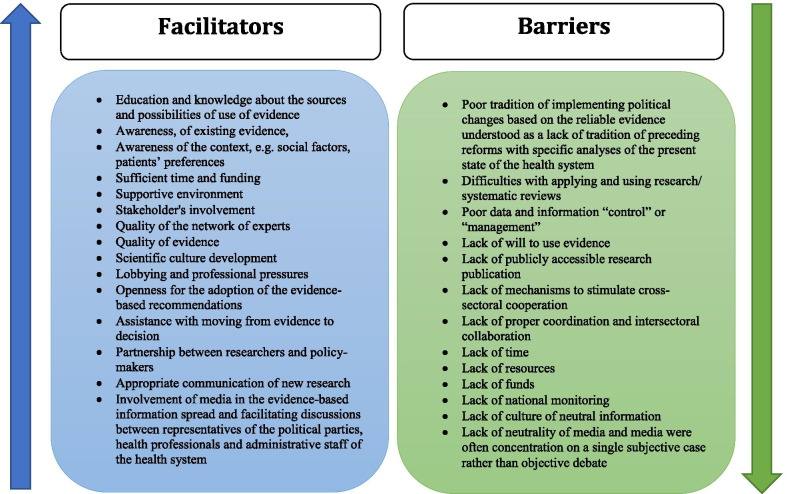
Facilitators and barriers in evidence uptake

Based on the responses of the CAs, we created categories of codes and grouped them as main themes which describe elements that facilitate and impede the evidence-based approach in Europe (Fig. 2).

Amongst the facilitators, we identified the following categories: education of actors/stakeholders/public, awareness, funds, supportive environment, quality, lobbying, assistance with moving from evidence to decision, and finally scientific culture development, which can include all the components mentioned above.

Barriers were grouped as follows: difficulties with applying and using research/systematic reviews, poor data and information “control” or “management”, lack of will, lack of popularization achieved by scientific research, lack of mechanisms to stimulate cross-sectoral cooperation, lack of proper coordination and intersectoral collaboration, lack of time, lack of resources, lack of national monitoring, lack of culture of neutral and impartial information, and poor tradition of implementing political changes based on reliable evidence, which can include all the components mentioned earlier.

### Facilitators

Education is a key factor in evidence use in policy-making. Decision-makers should be knowledgeable about the sources and possibilities for use of evidence, which can be beneficial for the policy and decision-making process. Support of evidence-based practice and policy through education about evidence-based solutions was stressed by the Norwegian CA. In Denmark, “the Danish Health Authority offers method courses in working with national clinical guidelines. Such courses contain an introduction to and explain how to assess the risk of bias and other sources of uncertainty in studies and across studies. The purpose of these courses is to disseminate information and knowledge about the method and the work with the national clinical guidelines” (Danish CA). Finland proposed the use of a handbook of early intervention to implement evidence-based early support, care and parental skills tools in practice (Finnish CA).

Educative initiatives increase awareness about evidence use. In Portugal, “there is a general recommendation about increasing the recognition of research, related to health and to the definition of health needs, program implementation and program evaluation” (Portuguese CA). The Portuguese CA also stressed the importance of lifelong training as a measure which provides sustainability, and she highlighted the role of “professional life long training; support from evaluation agencies that may monitor implementations and feedback the public policies” (Portuguese CA).

It is not only the awareness of existing evidence that matters; awareness of the context—for example, the social factors or patients’ preferences—is also significant in facilitating evidence usage. The Bulgarian CA stressed that “the obvious truth is that the outcome of treatment of many chronic diseases depends on the effect of the medical measures, depends on the social environment in which these measures are undertaken. If these social factors are not taken into account, there is a high risk that the medical measures are not implemented” (Bulgarian CA). From the perspective of the Austrian CA, “elements which will facilitate evidence-based practice are, for instance, that objective facts which should be taken as decision support, (…) evidence should be seen as complementary by considering experience and patients’ preferences” (Austrian CA).

Both categories, education and awareness, can assist with moving from evidence to decision and recommendations—in other words, from evidence to policy-making. Evidence should be highly relevant to existing problems. The Norwegian CA recommended conducting systematic literature searches with the sorting of results (as a brief report) as an important starting point for a specific review.

The Irish CA stressed that, amongst the elements which can facilitate evidence usage, there might be a mixture of political orientation and access to funds with supportive staff aimed at providing evidence-based medicine and healthcare services within their given budget (Irish CA). This led us to identify two new categories of facilitators: funds and supportive environment.

Sufficient time and funding as a key factor in evidence use was stressed by the British CA, who noted that “clinical practitioners need to be encouraged to participate in research and so require time as well as funding to do this” (United Kingdom CA). This goes hand in hand with the financial possibilities of the formulation of a unit contributing to evidence-based decisions. Such an institution was created in Norway in 2011. Several Norwegian directorates established a “Unit for Social Welfare Research” whose mandate was to “contribute to evidence-based decisions about policy and practice in Norwegian welfare services, especially by conducting systematic reviews” (Norwegian CA).

The concept of a supportive environment refers to the support from evaluation agencies that may monitor implementation and feedback regarding public policies (Portuguese CA) and “the strong engagement of public services stakeholders (at national, regional and local level), and also third sector organisations (…) together with child rights advocacy groups and research and educational institutions” (Spanish CA). Performing consultancy work such as assistance with applications, research bids, assessing single studies, and research and development was significant from the perspective of the Norwegian CA. Amongst factors influencing the support of evidence uptake in policy and practice is the literacy of the involved stakeholders, as stressed by the Czech CA. He pointed out that “the involvement of the various Societies of the Czech Medical Association of J. E. Purkyně: Czech Society of Adolescent Medicine, Czech Endocrinology Society, Czech Society for Obesitology, Czech Pediatric Society, Society of PLDD (GPs for Children and Adolescents)” is beneficial (Czech CA).

The Finnish CA stressed how consultation with experts can affect evidence-based policy-making. He noted that “the major decisions are made by politicians, but the civil servants usually ask expert opinions before decision-making. In Finland, the national and regional authorities with knowledge on child and adolescent health and services as well as evidence-based practices are frequently consulted. In this case, a larger scale project was initiated and it was given to THL, the National Institute for Health and Welfare, which is the leading institute in health and welfare services in Finland” (Finnish CA).

In addition to the importance of stakeholder involvement, the quality of the network of experts was mentioned by the Slovak CA. He proposed to “create expert working groups with these experts as well as significant authorities from public service involved” (Slovak CA) to support evidence use in his country. The Polish CA added that access to highly qualified scientific staff and high-quality education in medical professions are significant facilitators as well. In Romania, it is important that “correct information should always come from professionals who can provide scientific evidence” (Romanian CA).

This would consequently affect the quality of evidence and encourage its use. The Norwegian CA added that “further research development work in summarizing qualitative studies” (Norwegian CA) is a crucial facilitator as well. Another facilitator of evidence use worth stressing is the development of “evidence for informed decision-making on the effect of interventions in social welfare services by conducting systematic reviews and other secondary research—by producing plain-language summaries of existing systematic reviews” (Norwegian CA).

Lobbying and professional pressure were mentioned by Croatian and Polish CAs as elements which can significantly affect the design, uptake and implementation of evidence-based solutions.

Quality improvement is an important element of scientific culture development. An Italian expert added that “schools should improve the scientific culture of pupils. Citizens should take greater account of the scientific preparation of people who they are willing to elect. The ways of selecting political representatives should be changed and improved” (Italian CA).

Openness to the adoption of evidence-based recommendations is another component of scientific culture development. In Greece, “significant improvements have been made in the last few years, particularly in the area of medical prescriptions. For example, regarding the management of chronic diseases, evidence-based national guidance and management protocols have been adopted and are mandatory within the e-prescription system” (Greek CA).

For building the scientific culture, collaborative partnership between researchers and policy-makers and appropriate communication of new (usually) synthesized research relevant to the directorates (Norwegian CA) is needed. Facilitating active collaboration and communication among welfare directorates in Norway seems to be crucial for supporting evidence usage.

### Barriers

One of the barriers which we observed based on analysis of the data provided by CAs was a very poor tradition of implementing political changes based on reliable evidence, which can be interpreted as a lack of tradition of previous reforms with specific analyses of the present state of the health system—as reported by the Polish CA. Additionally “short experience in the field of science, business and politics” (Polish CA) was stressed.

The Norwegian CA considered as a challenge difficulties with applying and using systematic reviews to inform policy. In Croatia, data and information “control” or “management” nationally needs to be improved, with the goal of focusing on “evidence-based policy-making” (Croatian CA). The CA from Cyprus also stressed that there is a lack of will to use evidence in the policy-making process, and it can significantly affect the use of evidence.

The resistance of the medical environment before implementing changes was also highlighted as a barrier by the Polish CA. Additionally, lack of popularization of achieved scientific research and lack of mechanisms to stimulate cross-sectoral cooperation were also mentioned as difficulties in applying evidence-based policy-making in practice. This is closely correlated with lack of proper coordination and intersectoral collaboration highlighted by the Greek CA. In Greece, this problem is linked with “differences in the legislation of public health care regarding administrative work, unbalanced funding and expense cuts, unclear job descriptions and relationships and cases of conflicting interests” (Greek CA).

The consequence of a lack of interest in evidence-based practice in child healthcare and healthcare policy can be lack of time or lack of resources, with the result that “trajectories do not run smoothly, and it happens that some actions are initiated or finished without a proper evaluation” (Portuguese CA). Lack of funds disrupts the adequate monitoring of actions and impedes the training of professionals (Portuguese CA). The Danish CA stressed that evidence-based initiatives require a resource-intensive approach. He noted that “an element that impedes the approach to evidence-based practice is that evidence-based practices are resource-intensive and require the employees to have strong competencies” (Danish CA). Another factor pointed out by the Danish CA was a lack of national monitoring of whether the guidelines were implemented and whether they were followed.

Lack of a culture of neutral information was observed by the Italian CA, who noted that “the habit of discussing on the basis of the opinion of authoritative experts is still deeply rooted. The culture of ‘neutral’ (as much as possible) information is lacking” (Italian CA).

### Twofold media impact: facilitator and barrier effect

Use of evidence is strongly embedded in context. It was proved by the responses of CAs, which show that media can both facilitate and impede the development of the new health policies.

The Austrian CA stressed that several discussions took place in conferences such as the European Forum for Evidence-based Health Promotion and Prevention, when the new mother and child screening was implemented. However, she also emphasized that “it is also not always possible to meet all criteria of the evidence-based medicine to one hundred percent. In some subjects, this is also difficult—for example, in paediatrics” (Austrian CA). In Croatia, it was the starting point for the vast majority of communications and discussion with the public when the national children’s amblyopia plan was introduced. Many countries reported that the information about the evidence used in the policy-making process was widely present in the media. This played a significant role in the broadcasting and distribution of evidence-based approaches in European countries.

Media as facilitator in the discussions was mentioned by the Greek CA, who said that “results and suggestions by the report were used mainly through news media (TV and printed press), in facilitated discussions between representatives of the political parties, health professionals and administrative staff of the health system” (Greek CA).

Media, including social media, also played a powerful role in Romania in influencing public opinion, when the new law on vaccination was drafted. However, the Romanian CA stressed that the media input did not necessarily, or did not always, produce accurate or positive messages. A lack of neutrality was expressed. According to the Romanian CA, at the beginning of the vaccine crisis, both state and private television invited at least one anti-vaccination physician or active anti-vaccination campaigner on social media to give their views. Most of the time, these people participated alone in shows, without the opportunity for debate or discussion of the plurality of opinions. These shows had a large audience and were broadcast at times when they would reach the maximum audience. The most challenged vaccines were measles and polio, which were presented as having dangerous health consequences; others such as the BCG (bacillus Calmette-Guérin) vaccination were considered to have no positive effects, serving only the interests of pharmaceutical companies, who were portrayed as only interested in profit. At a later stage, when measles epidemics occurred, shifts in risk perception were noticeable in the media. The national media became an important actor advocating in favour of vaccination: “Journalists from TV and print media turned their campaign around and reported illness and death, and pressured governments to take concrete measures to limit the epidemics. These pressures forced the Health Ministry to clarify through press releases and new legislative regulations to avoid the vaccine crisis, whose priority cause is the lack of dose requirements for populations entering the national vaccination scheme” (Romanian CA).

It was also reported that the media were often focused on a single subjective case rather than objective debate. Lack of neutrality was identified by the Italian CA, who mentioned that “media often prefers to present a single pathetic case (e.g. a baby died due to meningitis, or another has become autistic after a vaccination), instead of putting on a fair and objective debate (which would take a long time)” (Italian CA). Conversely “in the very intense public debate on media, expert opinions have been reported: ‘pro vs con's’ style. Probably, there was a lack of presentation of evidence from literature, especially for healthcare professionals” (Italian CA). The Polish CA added that “the provisions of the Act of Primary Health Care differ significantly from those presented by the media. The main changes have not been fully presented, and the media has focused only on certain elements. The media was presenting as especially controversial the fact that paediatricians were not included in the primary care team in the provisions of the Act (which was later changed). The critical voices of experts at the professional media portals dominated the discussion. Emotional media reports of tabloid media have also been reported, which caused alarm that children's health is at risk” (Polish CA).

On the other hand, the Croatian national “Healthy Living” programme was also “highly visible in mass and social media in various forms including commercials and organising and visiting events and shows where the information, background and evidence together with education have been provided and awareness continuously raised” (Croatian CA). Similarly in Romania, where the high death rate among measles cases “has turned a large part of the media in favour of the vaccination (…) as it has consistently presented the victims of the measles epidemic and has put pressure on the health ministry to explain and find a solution” (Romanian CA), media played an educative role in increasing awareness in the population. However, the Portuguese CA stressed that “media may highlight issues, but public policies are not dependent on media news but rather from identified needs”.

## Discussion

Recognizing the importance of evidence-based solutions in policy-making is crucial for effective implementation of innovation within child health policy. In the process of evidence-informed policy-making, understanding who is producing evidence is important. WHO stresses that “expert opinion is more than just evidence” [5] because it contains facts, their interpretation and conclusions [5]. Research evidence is considered more reliable than expert opinion because it “uses systematic methods to collect and analyse observations” [5]. Therefore, we sought to identify the level of research evidence that underpins national policy in EU and EEA countries.

Our study identified a few generic factors that may influence the efficiency and success of child health policy. We were particularly interested in the facilitators and barriers in the use of evidence in child health policy-making in Europe.

We observed two key trends in potential evidence-based knowledge transition in policy-making. On the one hand, the importance of academia and its achievements was stressed, and on the other hand the role of institutional reporting was highlighted. We defined those types of approaches as scientific and institutional. The scientific approach comprises so-called bottom-up initiatives from the professional scientific environment, whereas the institutional approach involves top-down evidence production, which is initiated by administrative institutions. We found that the institutional approach seems to benefit from the scientific resources and presents its recommendations based on scientific achievements; thus the two groups are not mutually exclusive. This was also discussed by Cairney and Oliver [22], who reported that “successful engagement in ‘evidence-based policy-making’ requires pragmatism, combining scientific evidence with governance principles, and persuasion to translate complex evidence into simple stories” [22].

The evidence used in Europe can also be described as of an external or internal nature. Such classification is consistent with the WHO approach, which considers evidence through both global and local prisms [3]. International comparisons play a significant role as a contextual determinant of child health policy-making [20]. They facilitate the exchange of good practices and provide information that is not always available nationally. Domestic evidence, on the other hand, is strongly contextualized. By definition, it devotes attention to nationally sensitive issues which need to be resolved. Nationally produced data are a source of country-specific evidence, which should be crucial in national policy-making processes. Successful policy-making seems to involve a combination of international evidence that is applicable to nationally identified issues. This was seen as one of the key points of the Organisation for Economic Co-operation and Development (OECD) conference “Governing better through evidence-informed policy-making” in 2017 [23]. The discussion on better leverage and connectedness of the existing international networks that exist to improve their effectiveness and diffuse their result raised the question, “how can international networks for evidence create results that are relevant at the local level?” [23]. Our results address this question by indicating the means of evidence production at both levels as well as the direction of the relationship between them (Fig. 1).

Hasanpoor et al. highlighted that “the practical framework of evidence-based management should be based on the best resources from identifying barriers and facilitators” [9]. In our study we described both of these.

We found that various factors can affect evidence use in child health policy-making. Head, in 2010, pointed out three elements that underpin modern conceptions of evidence-based policy: (i) high-quality information based on relevant topic areas, (ii) professionals with skills in data analysis and policy evaluation, and (iii) political incentives for using evidence-based solutions in governmental decision-making processes [8]. Our analysis of facilitators and barriers shows that policies are more likely to be transferable if they meet these three criteria for success. In our study we highlighted the significance of the quality of the policy processes and quality of information, need for the development of a tradition of implementing political changes based on reliable evidence and the demand for a supportive environment for evidence-based policy-making.

This was also found by Bach-Mortensen et al. [24], who noted that the most frequently reported facilitating factors in their study were related to whether the evidence-based intervention aligned with the mission of the organization, the flexibility to implement the evidence-based intervention, its perceived effectiveness and its organizational support and prioritization, and finally, supportive leadership. Our data show that lobbying groups have particular importance in the process of creating supportive environments, because they bring implementation of financial support to generate high-quality evidence.

We found that barriers to evidence-based policy-making include a poor tradition of implementing changes based on reliable evidence, which is also reflected in the paper by Bach-Mortensen et al. [24] as a set of determinants which give rise to dysfunctional organizational culture. Where there are difficulties with the application and use of research evidence in policy, it tends to be because of poor data and information control or management. In the literature it is reflected in barriers related to policy-maker research skills [25] or data management issues [24]. The reasons identified in our study include a lack of will amongst decision-makers to use such data, but also a lack of funds to support evidence-based solutions in policy-making. These elements are consistent with the work of Ellen et al. [26], who described limited resources and negative attitudes as key barriers to implementing support for evidence-informed decision-making in health systems. In our understanding, in order to overcome these barriers, activation of mechanisms to stimulate cross-sectoral cooperation and proper coordination of intersectoral collaboration are required.

**Fig. 3:**
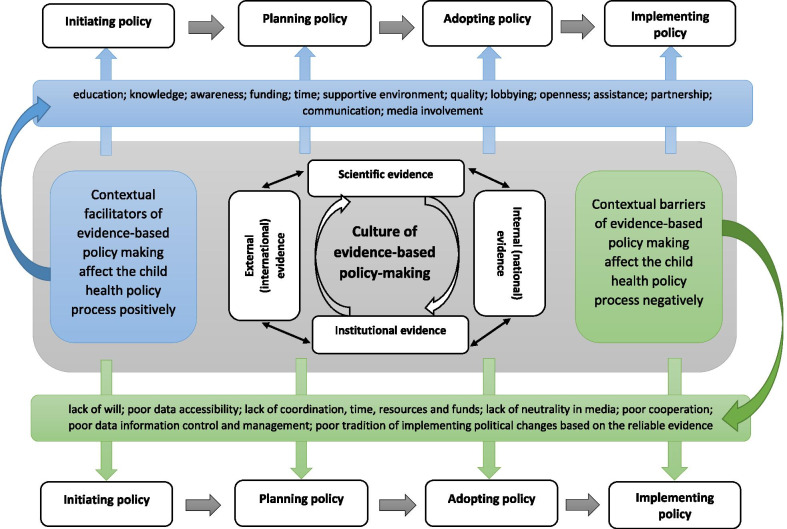
Culture of evidence-based policy-making

Generic elements that were identified which affect evidence-based solutions in policy-making constitute the phenomenon of a “culture of evidence-based policy-making” (Fig. 3), which can be interpreted as a style of policy-making where evidence is fundamental. Other authors have highlighted similar concepts in which knowledge translation culture [26] is understood as a “paradigm to address many of the challenges in translating research to knowledge users and start closing the ‘know–do’ gap” [26, 27].

In our understanding, the culture of evidence-based policy-making is broader, as it highlights not only challenges for the successful translation of evidence, but also explains the levels of evidence usage and shows the correlation between them. It is important to appreciate that the culture of evidence-based policy-making exists in parallel with the policy-making cycle. It is based on the appreciation of the value of scientific research which supports and draws from institutional data. These data can be expressed at two levels: national, which reflects the internal approaches to evidence-based policy-making, and international, which expresses external views. The culture of evidence-based policy-making is strengthened by facilitators and weakened by barriers.

## Limitations

The study on evidence usage in child health policy-making in European countries is challenging, as evidence usage is strongly contextualized. Some elements such as perceptions and attitudes are often internalized and introspective, making their influences difficult to determine.

In our research we tried to identify the types of evidence used and the factors which facilitate or impede evidenced-based policy-making. Although the MOCHA project had 30 national experts, we received responses from only 23 countries, as seven CAs did not provide responses to our questions. However, the nature of our research was not quantitative but qualitative; hence omission of some countries does not invalidate the data received. Our goal was to qualitatively investigate the key generic factors influencing evidence-based policy-making and its impact on child health policy.

### Practical implications

In this study we analysed factors which facilitate and impede evidence-based solutions within the context of child health policy in any country. We proposed guidance for stimulating effective reform processes with the goal of making the current factor analysis available to those who might wish to use it in their own reform process (Fig. 3).

Evidence-based child health policy-making needs an appropriate culture of evidence-based policy-making. Evidence usage is fundamental here; hence the creation of new policies and/or reforming current policies requires appropriate approaches based on available, high-quality evidence (Fig. 1). This might be scientific evidence produced by academic experts who are identifying existing problems that need to be solved and proposing appropriate steps to be undertaken. On the other hand, the evidence may come from institutions responsible for the reform or the introduction of new policy, or from analysis of problems with existing services. Representatives of national governments or other expert units might initiate research, studies or analyses in the areas where potential change is needed. Evidence might be sought nationally or internationally.

Evidence usage in policy-making may be facilitated or impeded. The characteristics of both types of elements were presented previously (Fig. 2). An environment conducive to the use of evidence in child health policy-making strengthens the culture of evidence-based policy-making, whereas lack of such an environment most probably will result in impeding a culture of evidence-based policy-making.

With this in mind, we would like to propose the following guidance for decision-makers which we hope will facilitate and promote the culture of evidence-based policy-making in their countries.

- Scientific evidence identifies and guides actions which need to be undertaken in the appropriate area.
- Institutional evidence relates to problems which have already emerged and need response from national or local institutions.
- Policy-making based on evidence usage truly responds to the population needs, identified scientifically and/or institutionally.
- Understanding that the facilitators of and barriers to evidence-based policy-making which we identified in our paper affect policy effectiveness.

## Conclusions

A culture of evidence-based policy-making is understood as a style of policy-making where evidence is fundamental. We identified a number of barriers and facilitators in the use of evidence to support policy development or policy change. Focusing on the facilitators and actively working to reduce the barriers can potentially lead to faster and more robust policy-making, including the development of a culture of scientific grounding in policy creation.

- Two types of evidence usage exist: scientific and institutional. The first one we understand as initiatives of the professional scientific environment; the second refers to evidence production initiated by administrative institutions.
- The evidence used in Europe can be of an external or internal nature.
- Various factors can affect the use of evidence in child health policy-making. Facilitators are correlated with strong scientific culture development, whereas barriers are defined by a poor tradition of implementing changes based on reliable evidence.
- Media are an important influence on the culture of evidence-based policy-making by facilitating or impeding evidence usage.
- The phenomenon of a culture of evidence-based policy-making is a style of policy-making where evidence is fundamental.
- A culture of evidence-based policy-making highlights challenges for the successful translation of evidence into policy, explains the levels of evidence usage and shows the correlation between them.

## Supplementary Information

**Additional file 1:** Questionnaire.

## Data Availability

Under Horizon 2020 funding rules, the European Commission requires all data to be accessible to the greatest degree possible, while also recognizing the potential conflict between openness and confidentiality. To that the MOCHA team have added the importance of comprehension, as source data cannot be interpreted without a clear understanding of the method of acquisition. The MOCHA data contain no patient information, but may contain other personal or institutional data such as source of a commentary. The MOCHA project has therefore resolved that source data will be curated on the MOCHA website, and will be accessible via the Principal or other partners through a curator function, so that data relevant to any enquiry can be supplied, and redaction effected, but also contextualization given.

## Abbreviations

CA: Country agent
EU: European Union
EEA: European Economic Area
FGM: Female genital mutilation
MOCHA: Models of Child Health Appraised
MKP: Mother–child passport
MP: Member of Parliament
PENIA: National Strategic Plan for Childhood and Adolescence in Spain
