# Supplementary material for: Factors influencing the uptake of evidence in child health policy-making: results of a survey among 23 European countries
Source: Health Res Policy Syst. 2021 Nov 7;19:136. doi: 10.1186/s12961-021-00786-y (PMC8573924; doi:10.1186/s12961-021-00786-y)
Supplement: Supplementary file 1 — Additional file 1: Questionnaire. [file 12961_2021_786_MOESM1_ESM.docx]

# Round 12: WP1 Characteristics of content and processes of child health policy through the prism of contextual determinants

**Please send your answers to Denise Alexander by 4th July 2017**

***Note:*** *These are the items analysed out of a wider data collection exercise.*

## Glossary

**Policy -** Broad statement of goals, objectives and means that create the framework for activity. Often take the form of explicit written documents, but may also be implicit or unwritten (Buse & Mays, 2005).

**Health policy** – courses of action (and inaction) that affect the set of institutions, organisations, services and funding arrangements of the health system (Buse & Mays, 2005).

**Child health policy** – courses of action that refer to child health.

**The content of (child) health policy** – Substance of a (child) health policy which details its constituent parts (modified/adapted from Buse & Mays, 2005).

**The process of (child health) policy** – The way in which (child health) policies are initiated, developed or formulated, negotiated, communicated, implemented and evaluated. (modified/adapted from Buse & Mays, 2005).

**Contextual influences** are understood as the elements from proximal and distal child health care environment which relate to:

- **Socio-cultural factors** such as: broadly understood social environment (family, peers groups, community, school), lifestyle, social support, social interaction, living conditions, behavioural patterns, ethnicity, minorities issues, norms, values, symbols, beliefs, religion, knowledge etc.
- **Structural factors** such as: **political issues**, employment issues, demographic issues, technological issues, institutional issues, health care organization/delivery/services, economic infrastructure, trade, development etc..

or might be related to:

- **Specific event** which happens in your country unexpectedly such as environmental disaster, conflict or others such as local affair, planned/unplanned temporal event, planned/unplanned visit of important authority etc.
- **International situation or factors** such as wars, conflicts, cooperation between countries, introduction of new international law, migration wave etc.

**Types of evidence** (Bowi & Zwi, 2005)

- Research (Empirical evidence from randomised control trials and other trials, Analytic studies such as cohort or case-control studies, Time series analyses, Observations, experiences, and case reports, Qualitative studies, Before and after studies),
- Knowledge and information (Results of consultation processes with networks/groups, the Internet, Published documents/reports (including policy evaluations and statistical analyses),
- Ideas and interests Opinion and view—“expert knowledge” of individuals, groups, networks (shaped by past personal and professional experiences, beliefs, values, skills), Politics Information relevant to the agenda of government (Political risk assessment and saleability, Opportunity, Crises),
- Economics Finance and resource implications (Cost effectiveness or other forms of economic evaluation, Opportunity cost)

Please identify an example of up to **three** new policies set within the past three years (either currently in operation, or that are soon to be implemented), that affect child primary health care service provision. Examples of such policies may be: vaccination policy; services for children with special needs; approaches to dealing with abused children; mental health promotion in primary care; or changes to out of hours service availability

Other types of policy may concern general issues that are concerned with provision and access to care, human resources, or the organisation of the system of financing.

Please duplicate this questionnaire as many times as it is needed.

**Note**: *All the questions should be considered from the national level perspective. We are particularly interested in whether there was a response in the media or in popular culture (either positive or negative) to the policy decision that was made.* *In order to respond this question it might be useful to investigate the media discussions but also debates via official laws, regulations, procedures, administrative actions, incentives, or voluntary practices etc.*

### 1. Policy name

| Click here to enter text. |
| --- |

*Please state if the policy is currently operating or will be implemented soon. Please provide references to the appropriate policy documents*

| Click here to enter text. |
| --- |

### 7. In general terms, was any evidence used in formulating the policy?

7a. Please describe how was evidence introduced to clarify issues discussed in the wider media (e.g. TV, print media, social media etc.).

| Click here to enter text. |
| --- |

### 8. Was the policy decision generally accepted as ‘sensible’ based on the situation of your country?

Yes

No

Please make any further comments you feel are necessary:

| Click here to enter text. |
| --- |

### 9. What needs to be in place to support evidence uptake in policy and practice? Please describe the elements which facilitate and impede the approach to evidence-based practice in your country

| Click here to enter text. |
| --- |

**10. Do you have any further comments? Please note in the box provided**

| Click here to enter text. |
| --- |
